# Construction and comprehensive analysis of a ceRNA network to reveal potential prognostic biomarkers for lung adenocarcinoma

**DOI:** 10.1186/s12885-021-08462-8

**Published:** 2021-07-23

**Authors:** Lei Gao, Ling Zhang

**Affiliations:** 1 Department of Respiratory and Critical Care Medicine, The Second Hospital of Anhui Medical University, 678 Furong Road, Economic And Technological Development Zone, Hefei, 230601 Anhui Province China; 2 Department of Critical Care Medicine, The First Affiliated Hospital of Anhui Medical University, 218 Jixi Road, Shushan District, Hefei, 230022 Anhui Province China

**Keywords:** Circular RNA, Lung adenocarcinoma, Microarray expression, Competing endogenous RNA, Overall survival, Prognostic biomarker, Drug sensitivity

## Abstract

**Background:**

More and more studies have proven that circular RNAs (circRNAs) play vital roles in cancer development via sponging miRNAs. However, the expression pattern of competing endogenous RNA (ceRNA) in lung adenocarcinoma (LUAD) remains largely unclear. The current study explored functional roles and the regulatory mechanisms of circRNA as ceRNAs in LUAD and their potential impact on LUAD patient prognosis.

**Methods:**

In this study, we systematically screened differential expression circRNAs (DEcircRNAs), miRNAs (DEmiRNAs) and mRNAs (DEGs) associated with LUAD. Then, DEcircRNAs, DEmiRNAs and DEGs were selected to construct a circRNA–miRNA–mRNA prognosis-related regulatory network based on interaction information from the ENCORI database. Subsequently, the gene ontology (GO) and Kyoto Encyclopedia of Genes and Genomes (KEGG) pathway enrichment analysis were performed on the genes in the network to predict the potential underlying mechanisms and functions of circRNAs in LUAD. In addition, Kaplan–Meier survival analysis was performed to evaluate clinical outcomes of LUAD patients, and drug sensitivity analysis was used to screen potential biomarkers for drug treatment of patients with LUAD.

**Results:**

As a result, 10 circRNAs were aberrantly expressed in LUAD tissues. The ceRNA network was built, which included 3 DEcircRNAs, 6 DEmiRNAs and 157 DEGs. The DEGs in the ceRNA network of hsa_circ_0049271 enriched in biological processes of cell proliferation and the Jak-STAT signaling pathway. We also detected 7 mRNAs in the ceRNA network of hsa_circ_0049271 that were significantly associated with the overall survival of LUAD patients (*P* < 0.05). Importantly, four genes (PDGFB, CCND2, CTF1, IL7R) identified were strongly associated with STAT3 activation and drugs sensitivity in GDSC.

**Conclusions:**

In summary, a ceRNA network of hsa_circ_0049271 was successfully constructed, which including one circRNA, two miRNAs, and seven mRNAs. Seven mRNAs (PDGFB, TNFRSF19, CCND2, CTF1, IL11RA, IL7R and MAOA) were remarkably associated with the prognosis of LUAD patients. Among seven mRNA species, four genes (PDGFB, CCND2, CTF1, and IL7R) could be considered as drug targets in LUAD. Our research will provide new insights into the prognosis-related ceRNA network in LUAD.

**Supplementary Information:**

The online version contains supplementary material available at 10.1186/s12885-021-08462-8.

## Background

Lung cancer is the most common cause of cancer-related mortality worldwide. Lung adenocarcinoma (LUAD) is the leading subtype of all lung cancers, accounting for about 40 to 50% of all lung cancer cases [1]. Despite improvements in diagnosis and the considerable research into cancer therapy, the average 5 years survival rate of LUAD patients is still less than 20% [2], because most patients are usually diagnosed at late stages and have little opportunity for effective treatment [3]. Therefore, identifying a novel accurate and sensitive biomarker for the early diagnosis of LUAD patients is urgently needed.

Circular RNAs are a new type of endogenous noncoding RNA, derived from precursor mRNA back-splicing. Circular RNAs (circRNAs) have a circular covalently closed structure without 5′ caps and 3′ poly tails and higher tolerance to exonuclease digestion [4]. Compared to linear counterparts, circRNAs are highly stable and conservative [5], and which can be found in exosomes, plasma [6] and even in urine, and could be a new non-invasive biomarker [7]. Over the past few decades, these transcripts have been considered a by-product of RNA splicing and have long been ignored [8]. With the advent of high throughput sequencing technologies and development of computational technologies, thousands of circRNAs are detected in various organisms, particularly tumors [9]. Growing evidence demonstrated that circRNAs were involved in the initiation and development of cancer [10]. Recent studies proved that some circRNAs can act as a molecular sponge of miRNAs to regulate gene expression. For instance, up-regulating circRNA-MYLK can promote epithelial-mesenchymal transition in bladder cancer [11], CircHIPK3 acts as a miR-7 sponge to promote colorectal cancer growth and metastasis [12], circNRIP1 sponges miR-149-5p to promote gastric cancer progression [13], pro-metastasis process of circANKS1B in breast cancer [14]. Similarly, differentially expressed circRNAs were also identified in LUAD, such as hsa_circ_0020732 [15] and hsa_circ_0128332 [16] enhanced LUAD metastasis, hsa_circ_0006427 [17] suppressed LUAD progression, hsa_circ_0012673 [18] could promote LUAD proliferation, indicating that circRNAs play important roles in LUAD pathogenesis. Without a doubt, circRNAs could act as ceRNA by sponging miRNA to relieve the repression of miRNAs for their targets [19, 20]. Therefore, circRNAs may be potential biomarkers or therapeutic targets.

**Fig. 1:**
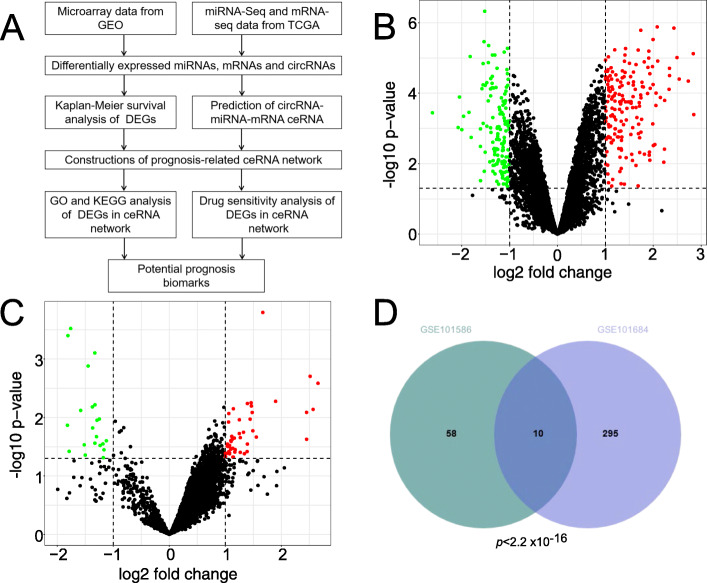
Volcano plot of differentially expressed circRNAs in LUAD. **A** Schematic plot for the identification and analysis of prognostic circRNAs in the ceRNA network (**B**) Differentially expressed circRNAs in GSE101684 dataset. **C** Differentially expressed circRNAs in GSE101586 dataset. **D** The overlap of circRNA transcripts detected in GSE101684 dataset and GSE101586 dataset was significant

In the present study, we have downloaded microarray datasets, GSE101684 [21], GSE101586 [22] and GSE29249 [23] from Gene Expression Omnibus (GEO, https://www.ncbi.nlm.nih.gov/geo/), the expression profiles of miRNAs and mRNAs in Cancer Genome Atlas (TCGA)-LUAD dataset. The expression levels of circRNA, miRNA and mRNA were comprehensively analyzed in LUAD. In order to identify the potential biological functions of the circRNA, differential expression of three kinds of RNA were screened to construct circRNA-miRNA-mRNA ceRNA network. Next, functional enrichment analysis was performed to reveal biological process and Jak-STAT signaling pathway of the ceRNA network in LUAD. The survival analysis demonstrated that seven mRNAs (PDGFB, TNFRSF19, CCND2, CTF1, IL11RA, IL7R and MAOA) regulated by circRNA (hsa_circ_0049271) were prognostic biomarkers in LUAD. Finally, there are some potential drugs which are specifically associated with mRNAs, indicating that effects of drugs in LUAD. Our findings suggested that the dysregulation of circRNAs in LUAD could be a novel diagnosis and treatment biomarkers (Fig. 1a).

## Methods

### Microarray data and RNA-Seq data

The circRNA and mRNA expression profiles in LUAD were obtained from GEO database. GSE101684 from the platform GPL21825, 074301 Arraystar Human circRNA microarray V2, which includes tumor samples and paired adjacent normal tissues from 4 patients. GSE101586 from GPL19978, Agilent-069978 Arraystar Human circRNA microarray V1, which provides five LUAD and five non-tumor adjacent lung tissue samples. GSE29249 from the platform GPL10558, Illumina HumanHT-12 V4.0 expression beadchip, which consists of six paired NSCLC cancer tissue and the adjacent normal tissue. Series Matrix File(s) and platform information were downloaded by ‘GEOquery’ R package. The expression levels of miRNAs and mRNAs in TCGA-LUAD dataset were also downloaded from the Cancer Browser (https://xena.ucsc.edu/welcome-to-ucsc-xena/) [24]. The detailed dataset information is described in (Table 1).

**Table 1:** Details for GEO and TCGA lung adenocarcinoma cancer data

Reference	Dataset	Platform	Normal	Tumor	RNA type
Zhao et al (2017) [21]	GSE101684	GPL21825	4	4	circular RNA
Chen et al (2019) [22]	GSE101586	GPL19978	5	5	circular RNA
Ma L et al (2011) [23]	GSE29249	GPL10558	6	6	mRNA
Goldman et al (2020) [24]	mRNA_HiSeq		45	450	mRNA
Goldman et al (2020) [24]	miRNA_HiSeq		45	450	miRNA

### Identification of differentially expressed transcript in LUAD samples

Log2 transformation was performed for each circRNA, miRNA and mRNA expression data. The probe name of circRNA, miRNA and mRNA were converted into circBase’s ID, mirBase’s ID and gene symbol respectively [25, 26]. The R package ‘limma’ in Bioconductor [27] or t test was used to implement circRNA, miRNA and mRNA differential expression. circRNA, miRNA and mRNA with the threshold set at a *P*-value < 0.05 and |log2-fold change (FC)| > 1 were considered as differentially expressed circRNA (DEcirRNAs), miRNA (DEmiRNAs) or mRNA (DEGs). A hierarchical cluster heatmap based on Euclidean distance was generated using the ‘pheatmap’ R package.

### Network analysis of ceRNA and function annotation

CircRNAs can function as miRNA sponges to bind to miRNAs and modulate downstream target genes of miRNAs. Based on the ENCORI database (http://starbase.sysu.edu.cn/), miRNA-circRNA pair and miRNA-mRNA pair information were obtained. To improve the ceRNA network reliability, DEcirRNAs, DEmiRNAs and DEGs were chosen to construct the ceRNA network. Additionally, miRNAs are negative regulators of gene expression, therefore, expression of the miRNA and mRNA in a ceRNA network should be negatively correlated, and expression of the circRNA and mRNA in a ceRNA nework should be positively correlated [28]. The ceRNA network was visualized using the ‘ggalluvial’ R package. To obtain a more comprehensive understanding of the potential mechanism and biological function of the ceRNA network, we implemented Gene Ontology (GO) and Kyoto Genome Encyclopedia (KEGG) [29] pathway analyses using the GESA online analysis database for DEGs in the ceRNA network [30]. The significant enrichment GO term and KEGG pathway were selected at a *P* value < 0.05.

### Survival analysis

The survival information of LUAD samples were downloaded from the Cancer Browser. After combining the overall survival of patients and the expression of DEGs in the ceRNA network, the ‘survival’ and ‘survminer’ R package were used to implement a survival analysis and draw Kaplan–Meier plot. The patients were classified into two groups (high vs. low) using median expression levels of DEGs. Log-rank *P* < 0.05 was considered significant.

### Drug–DEGs associations across cancer cell lines

GSCALite is a web-based analysis platform for gene set cancer analysis [31]. To investigate the effects of drug sensitivity on DEGs expression, GSCALite web tool was used to perform drug analysis. DEGs as input gene identifiers were submitted online for gene set analysis. Then the Spearman rank correlations were calculated between DEGs expression levels and IC50 values of drugs sensitivity across cancer cell lines in GDSC [32, 33]. Correlations with false discovery rate less than 0.05 were considered as significant pairs.

## Results

### Identification of differentially expressed circRNAs in LUAD

Microarray GSE101684 and GSE101586 dataset were re-analyzed to identify differentially expressed circRNAs in LUAD and matched adjacent normal tissues. There were 305 DEcircRNAs (168 upregulated DEcircRNAs and 137 downregulated DEcircRNAs) from GSE101684 dataset, which were shown in (Fig. 1b and Table S1). A total of 68 DEcircRNAs (47 upregulated DEcircRNAs and 21 downregulated DEcircRNAs) were obtained from GSE101586 dataset, which were shown in (Fig. 1c and Table S2). The cluster heatmaps of the top 10 DEcircRNAs for both datasets were shown in (Figure S1A and Figure S1B). The overlapped DEcircRNAs found in GSE101684 and GSE101586 dataset were significant (Fisher Exact Test *p* < 2.2 × 10–16) (Fig. 1d). In order to find key circRNAs that are dysregulated in LUAD, we choose 10 overlapped DEcircRNAs for further analysis. Finally, five upregulated (hsa_circ_0000519, hsa_circ_0003528, hsa_circ_0008274, hsa_circ_0072088 and hsa_circ_0082564) and five downregulated circRNAs (hsa_circ_0000662, hsa_circ_0003162, hsa_circ_0029426, hsa_circ_0043256 and hsa_circ_0049271) were identified as candidate circRNAs.

### Identification of differentially expressed genes in LUAD

**Fig. 2:**
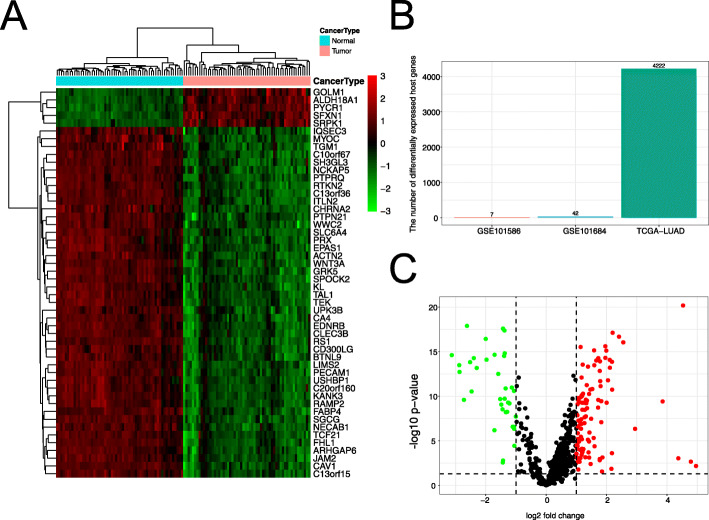
RNA expression profiles in LUAD and matched normal tissues. **A** Heatmap of the differential expression of the top 50 DEGs in TCGA-LUAD dataset. **B** The number of differentially expressed host genes associated with DEcircRNAs. **C** Differentially expressed miRNAs in TCGA-LUAD dataset

When GSE29249 dataset was screened by the ‘limma’ R package, 1742 differentially expressed genes (DEGs) were obtained. Among them, 932 upregulated genes and 810 downregulated genes were identified (Figure S2A and Table S3). In addition, 4222 DEGs were obtained from TCGA-LUAD, which included 1729 upregulated genes and 2493 downregulated genes (Figure S2B and Table S4). The cluster heatmaps of the top 50 DEGs were shown in (Fig. 2a). The overlapped DEGs in GSE29249 and TCGA-LUAD dataset were significant (Fisher Exact Test *p* < 2.2 × 10–16) (Figure S2C). Moreover, in order to investigate their prognostic value of DEGs regulated by circRNAs in LUAD, clinical information from TCGA-LUAD dataset were used.

Next, we analyzed the expression levels of circRNAs’ host genes in TCGA-LUAD, only 7 (0.17%) host genes associated with DEcircRNAs from GSE101586 dataset and 42 (0.99%) host genes associated with DEcircRNAs from GSE101684 dataset were found (Fig. 2b). These results indirectly suggested that expression levels of the circRNAs are not a reliable mediator of the corresponding abundance of host genes. Previous studies have demonstrated the average abundance of the host genes only weakly correlated with the average abundance of their associated circRNAs [4]. Another mechanism, that circRNAs can regulate genes in *trans* by absorbing microRNA, rather than induces host genes in *cis*. In other words, miRNA might play an important role in regulating ceRNA events in LUAD. Therefore, differentially expressed miRNAs (Fig. 2c and Table S5), circRNAs and genes were chosen to construct the network of ceRNA.

### Construction of a ceRNA network in LUAD

**Fig. 3:**
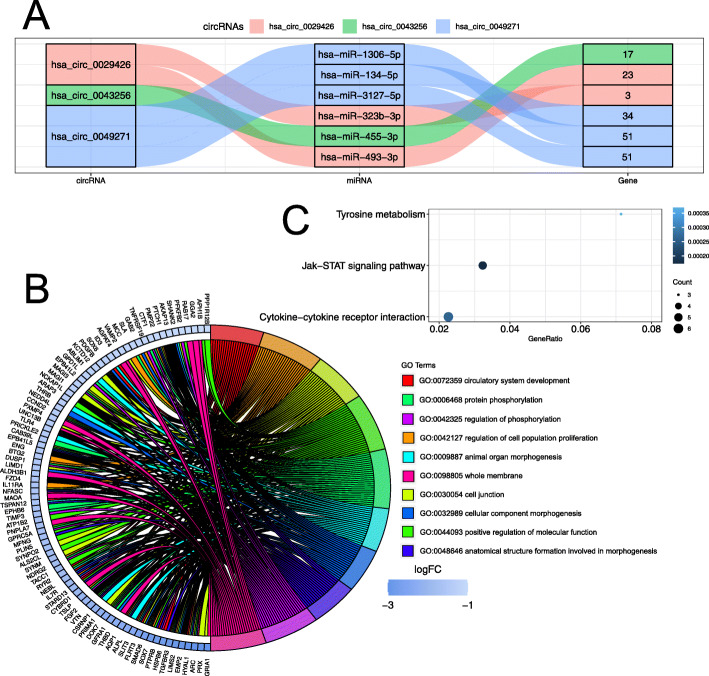
The ceRNA network in LUAD. **A** Sankey diagram for the ceRNA network of hsa_circ_0049271, hsa_circ_0029426 and hsa_circ_0043256. **B** Distribution of DEGs regulated by hsa_circ_0049271 in LUAD for different GO-enriched functions. **C** Significant pathway enrichment of DEGs regulated by hsa_circ_0049271 in the ceRNA network

In order to further understand the effect of circRNAs on mRNA regulated by combination with miRNAs in LUAD, we established the ceRNA network. The ceRNA network consisted of 3 (30%) circRNAs of 10 DEcircRNAs, 6 (4.08%) miRNAs of 147 DEmiRNAs and 157 (3.72%) unique mRNA of 4222 DEGs, which were visualized with the ggalluvial R package (Fig. 3a). Moreover, we observed 3 DEmiRNAs were bound to hsa_circ_0049271 (from GSE101684, log_2_FC = − 1.13, *P* < 1.70 × 10^− 4^, Table S1 and Table S6), which mediated 127 unique DEGs. In addition, we also found hsa_circ_0029426 (from GSE101684, log_2_FC = − 1.96, *P* < 4.06 × 10^− 3^, Table S1, Table S6 and Figure S3A) and hsa_circ_0043256 (from GSE101684, log_2_FC = − 1.39, *P* < 4.90 × 10^− 4^, Table S1, Table S6 and Figure S3B) sponging at least 1 different DEmiRNA to regulate 26 and 17 DEGs respectively. Collectively, these data suggested that hsa_circ_0049271, hsa_circ_0029426 and hsa_circ_0043256 may play important roles in LUAD.

### Functional enrichment analysis of DEGs in the ceRNA network

Gene ontology analysis with 127 DEGs regulated by hsa_circ_0049271 in the ceRNA network was conducted. The top 10 significant GO term results showed that hsa_circ_0049271 were divided into 2 groups, which consisted in cellular component and biological process groups (Table S7). These GO term results showed that hsa_circ_0049271 were mainly involved in biological processes of the progression of the circulatory system over time and any process that modulates the frequency, rate or extent of cell proliferation (Fig. 3b and Table S7). To identify the biological pathways related to hsa_circ_0049271, we conducted GSEA based on 127 DEGs. Then 3 pathways were identified, which were Cytokine-cytokine receptor interaction, Jak-STAT signaling pathway and Tyrosine metabolism (Fig. 3c and Table S8).

**Fig. 4:**
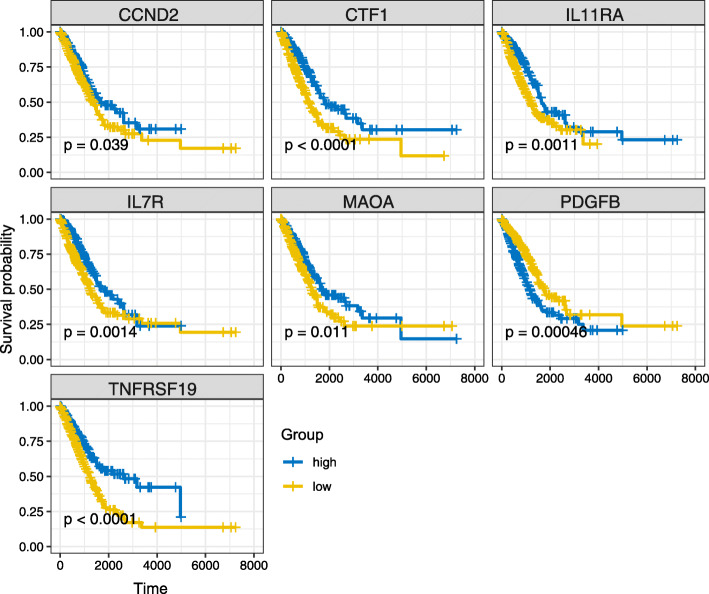
Kaplan–Meier survival curves of seven prognostic DEGs

**Fig. 5:**
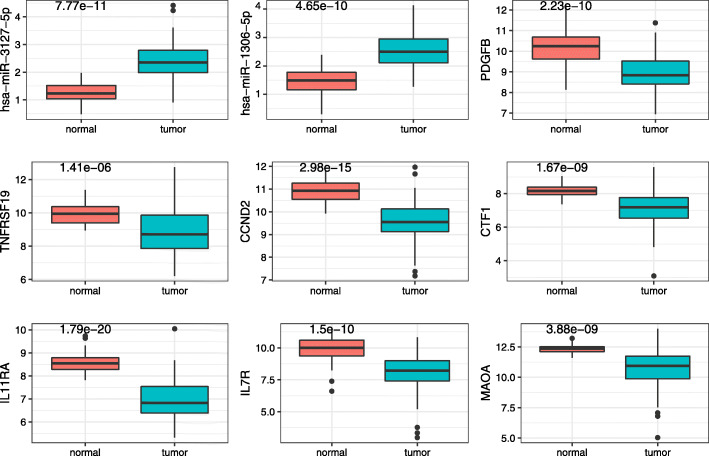
Differentially expressed transcript regulated by hsa_circ_0049271

We also analyzed expression profiles of their target genes in the significant KEGG pathway, and there were 7 prognostic risk target genes of hsa_circ_0049271 in the ceRNA network (Fig. 4). Finally, one circRNA (hsa_circ_0049271), two miRNAs (hsa-miR-3127-5p and hsa-miR-1306-5p), and seven mRNAs (PDGFB, TNFRSF19, CCND2, CTF1, IL11RA, IL7R and MAOA) were selected. The hsa_circ_0049271 was identified to be low expressed in LUAD, the decreased expression of hsa_circ_0049271, and the decreased expression of TNFRSF19, CCND2, CTF1, IL11RA, IL7R and MAOA in LUAD patients had a short OS period, which may be suppressor genes in LUAD patients. However, the decreased expression of PDGFB demonstrated a good prognosis and potentially could be a promoted cancer factor (Fig. 4 and Fig. 5). In addition, 15 DEGs regulated by other two circRNAs (hsa_circ_0029426 and hsa_circ_0043256) were significantly associated with clinical outcomes of patients with LUAD (Figure S4). These results showed that these genes were promising potential diagnostic biomarkers for LUAD patients.

### Contribution of DEGs as a predictor of anticancer drug response

**Fig. 6:**
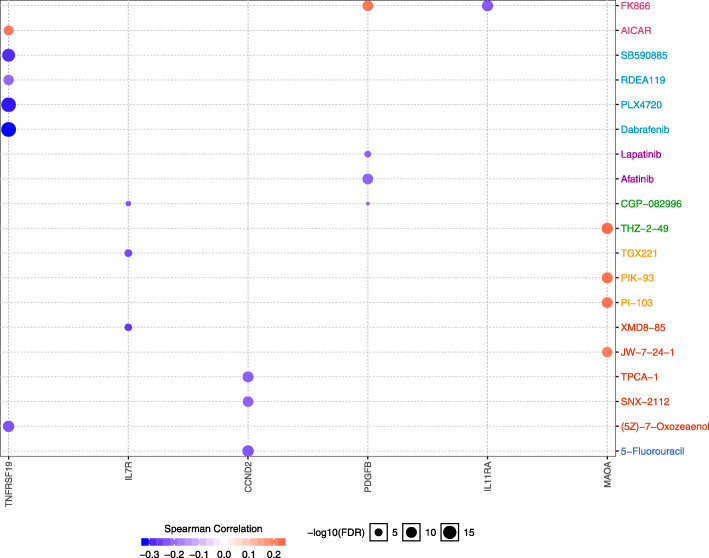
Contribution of DEGs as a Predictor of Anticancer Drug Response

To identify drug-related DEGs in LUAD, we thus explored the website GSCALite, the above seven target genes were selected for drug sensitivity analysis to find some potential drugs. The drug-DEGs associations results were obtained (Fig. 6). Analysis of these drug–DEGs associations, we observed that IL11RA was associated with one drug, while the expression of TNFRSF19 was associated with six drugs, suggesting their wide therapeutic effects. These results revealed that contribution of DEGs as a predictor of anticancer drug response.

## Discussion

Approximately 2.1 million new cases and 1.8 million deaths of lung cancer occur each year, making LUAD the cause of cancer incidence and mortality worldwide [34]. The early onset of LUAD is hard to screen, which leads to most of LUAD patients are usually found in the advanced stages of the disease. Therefore, Elucidating the molecular mechanisms of the tumorigenesis of LUAD is very important for identifying novel therapeutic targets and improving the clinical outcomes of LUAD patients. More and more studies have showed that circRNAs can serve as biomarkers for cancer diagnosis [35]. Compared to the traditional biomarkers, circRNAs are highly stable and conservative. Growing experimental evidence have proven that some circRNAs play important roles in the occurrence and development of LUAD acting as ceRNA [17, 36, 37]. The ceRNA hypothesis is a novel mechanism of RNA regulation by competing for shared miRNA response elements and provides important theoretical guidance for further understanding the tumorigenesis mechanism. However, the expression pattern of ceRNA in LUAD have not been thoroughly elucidated.

In our study, two microarray expression profile datasets from GEO dataset were used to identify DEcircRNAs in LUAD. Ten DEcircRNAs (5 upregulated and 5 downregulated) were significantly overlapped in two datasets, indicating important roles of circRNAs in LUAD. Moreover, hsa_circ_0000519 (circRNA-002178), hsa_circ_0072088 were identified, which were significantly upregulated in LUAD, and JunFeng Wang et al. revealed that circRNA-002178 could act as a miRNA sponge to promote PDL1/PD1 expression in LUAD [38], Liming Liang et al. confirmed that hsa_circ_0072088 were found differentially upregulated expression in LUAD with matched normal tissues by qRT-PCR with a *P* value < 0.05 [39]. However, circRNA-002178 was not in our ceRNA network based on our criteria (see methods). Because, we found that hsa-miR-34 regulated by circRNA-002178 was highly expressed in TCGA-LUAD tumor tissuesin our study and website tool (http://bioinfo.jialab-ucr.org/CancerMIRNome/) [40]. It was not consistent with previous result Wang et al. reported that hsa-miR-34 significantly downregulated in LUAD cancer tissues. We chose the same miRNAs expression profiles from TCGA-LUAD dataset as Wang et al’s. We analyzed the miRNA differential expression profiles of the 450 LUAD tissues and 45 non-tumor tissues. However, Wang et al’s study did not provide the TCGA samples number and samples name of LUAD tissues and non-tumor tissues they used. Therefore, we guessed the inconsistent result due to different selection of tissue samples, and excluded circRNA-002178 ceRNA network for further analysis. In addition, hsa_circ_0043256 was significantly downregulated in LUAD. As prior study showed that hsa_circ_0043256 could inhibit cell proliferation and induced apoptosis in A549 and NCI-H460 cells [18]. These findings suggest that our bioinformatics method is valid for identifying potential function of circRNAs.

To investigate the roles of circRNAs in LUAD, we analyzed the expression profiles of their host genes in TCGA-LUAD samples. However, only very few genes were differential expression genes. Thereby, we hypothesis that circRNAs have more widely impact on transcripts via sponging miRNAs. Based on the ceRNA hypothesis, 10 DEcircRNAs, 147 DEmiRNAs and 4222 DEGs were selected to construct the circRNA-miRNA-mRNA regulatory network, and we found that hsa_circ_0049271 interacted with most nodes. The GO enrichment analysis indicated that the function of 127 DEGs regulated by hsa_circ_0049271 could be involved in cell proliferation. Through KEGG pathway analysis, Cytokine-cytokine receptor interaction (map 04060), Jak-STAT signaling pathway (map 04630) and Tyrosine metabolism (map 00350) were detected, which are cancer-related pathways. The Jak-STAT signaling pathway is significantly involved in the development of solid tumors [41]. G Guney Eskiler et al. showed that IL-6 mRNA levels considerably increased 5.18 ± 2.81-fold in lung cancer, and the changes in the STAT3 and IL-6 expression levels may mediate STAT3 activation [42]. In addition, crizotinib reported by Anh et al. [43] and Hongmin Lu et al. [44] can promote apoptosis and inhibit cell proliferation of cells through JAK-STAT pathway in LUAD. Interestingly, the expression levels of four genes (PDGFB, CCND2, CTF1, IL7R) identified in the current study were strongly associated with STAT3 activation, clinical outcome of LUAD patients and drugs sensitivity across cancer cell lines in GDSC. All these above views indicated that the genes related to Jak-STAT signaling pathway reflects vital mechanisms of LUAD.

The expression levels of circRNA, miRNA and mRNA were comprehensively analyzed in TCGA-LUAD dataset. However, several limitations must be noted. First, DEcircRNAs identified in the present study were limited by sample size. Second, our findings need to be validated experimentally, which would be carried out in our following study.

## Conclusions

In summary, a ceRNA network was successfully constructed, which including one circRNA, two miRNAs, and seven mRNAs. Seven mRNAs (PDGFB, TNFRSF19, CCND2, CTF1, IL11RA, IL7R and MAOA) were remarkably associated with the prognosis of LUAD patients. Among seven mRNA species, four genes (PDGFB, CCND2, CTF1, and IL7R) could be considered as drug targets in LUAD.

## Supplementary Information

**Additional file 1.**

**Additional file 2.**

**Additional file 3.**

**Additional file 4.**

**Additional file 5.**

**Additional file 6.**

**Additional file 7.**

**Additional file 8.**

**Additional file 9.**

**Additional file 10.**

**Additional file 11.**

**Additional file 12.**

**Additional file 13.**

## Data Availability

The authors declare that the data supporting the findings of this study are available within the article. The datasets generated and/or analysed during the current study are available in the GEO repository, https://www.ncbi.nlm.nih.gov/geo/. The RNA-seq data of raw and processed files have been deposited in NCBI Gene Expression Omnibus, accession number GSE101684, GSE101586 and GSE29249. All TCGA related data can be obtained from the TCGA database (https://tcga-data.nci.nih.gov/).

## Abbreviations

circRNAs: circular RNAs
LUAD: Lung adenocarcinoma
GO: Gene ontology
KEGG: Kyoto Encyclopedia of Genes and Genomes
ceRNA: Competing endogenous RNA
GEO: Gene Expression Omnibus
TCGA: The Cancer Genome Atlas
