# Supplementary figures and images for: Construction and comprehensive analysis of a ceRNA network to reveal potential prognostic biomarkers for lung adenocarcinoma
Source: BMC Cancer. 2021 Jul 23;21:849. doi: 10.1186/s12885-021-08462-8 (PMC8299662; doi:10.1186/s12885-021-08462-8)

A

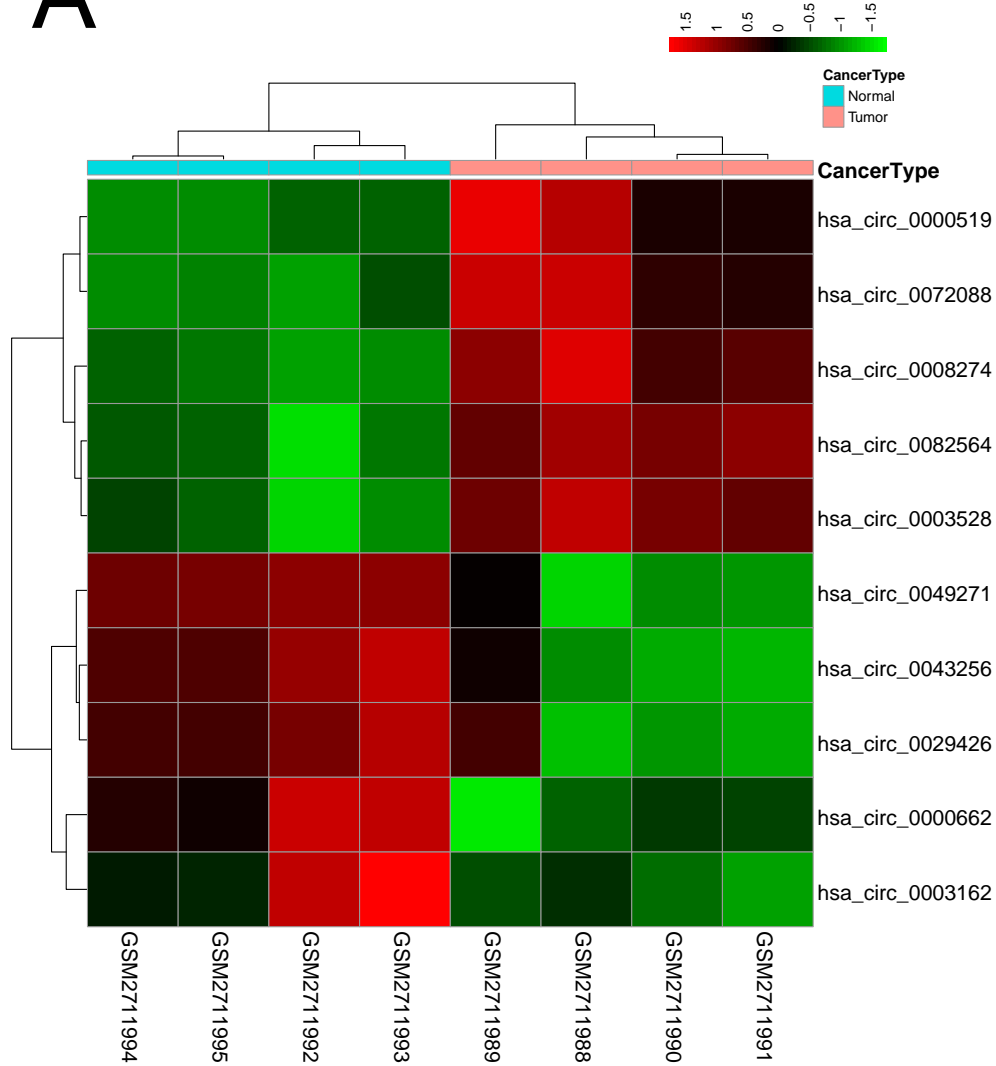

B

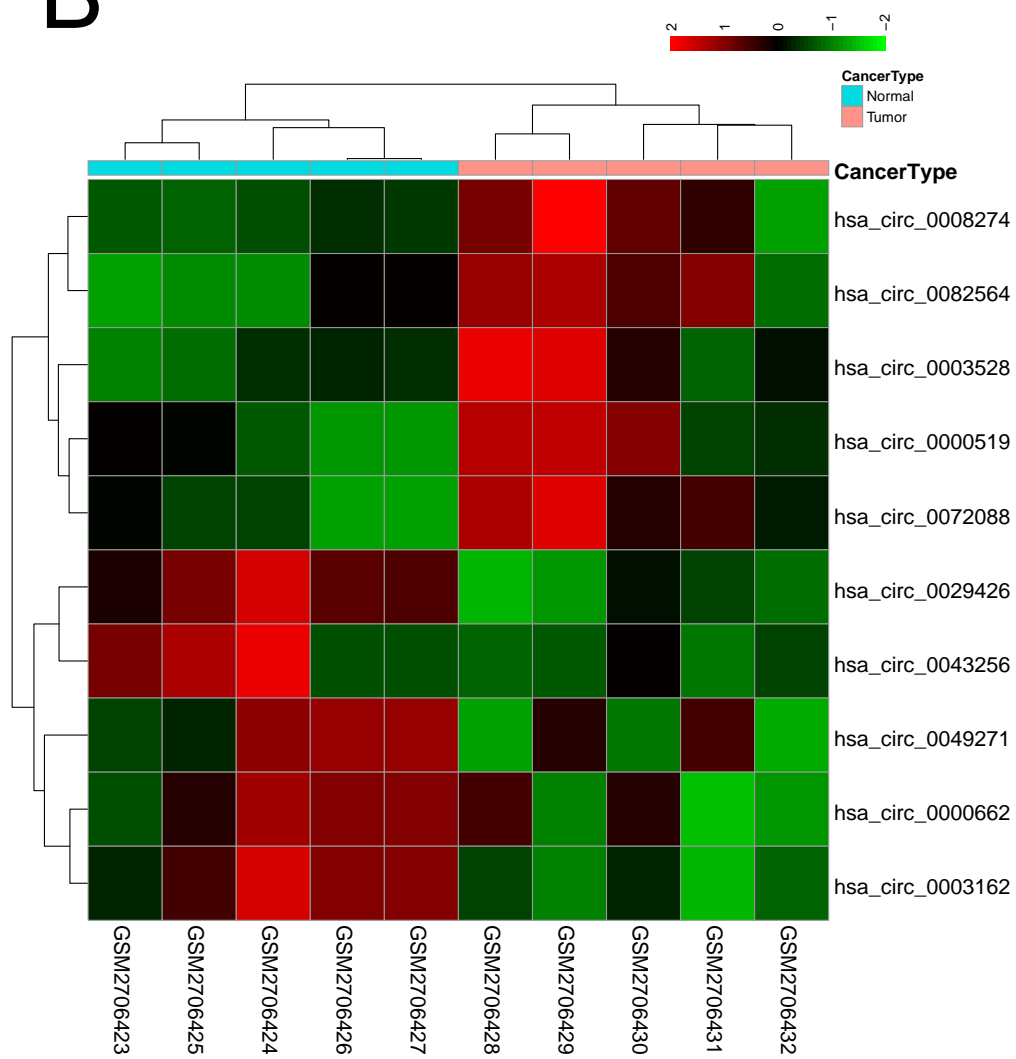

Supplement: Supplementary file 9 — Additional file 9. [file 12885_2021_8462_MOESM9_ESM.pdf]

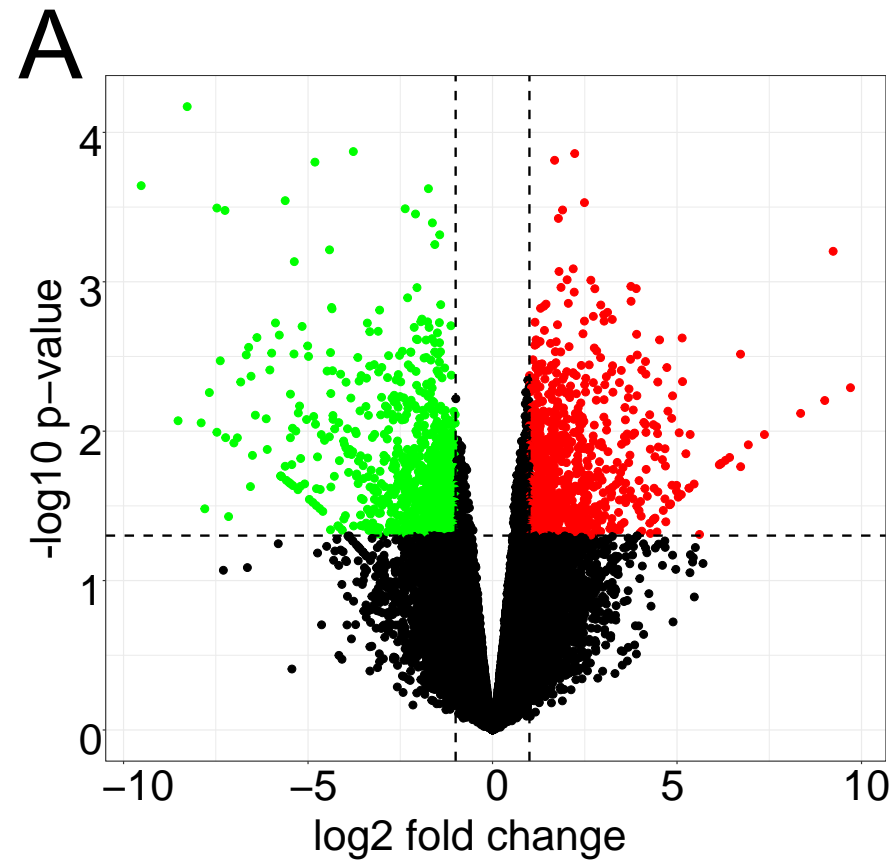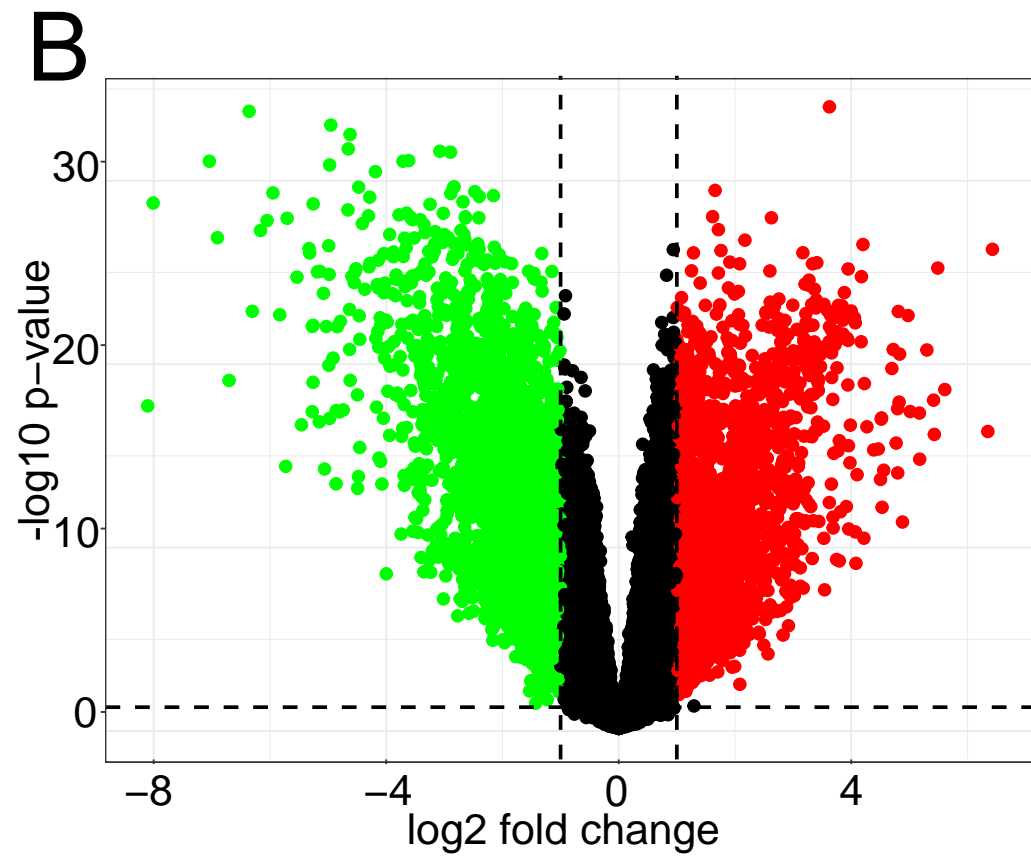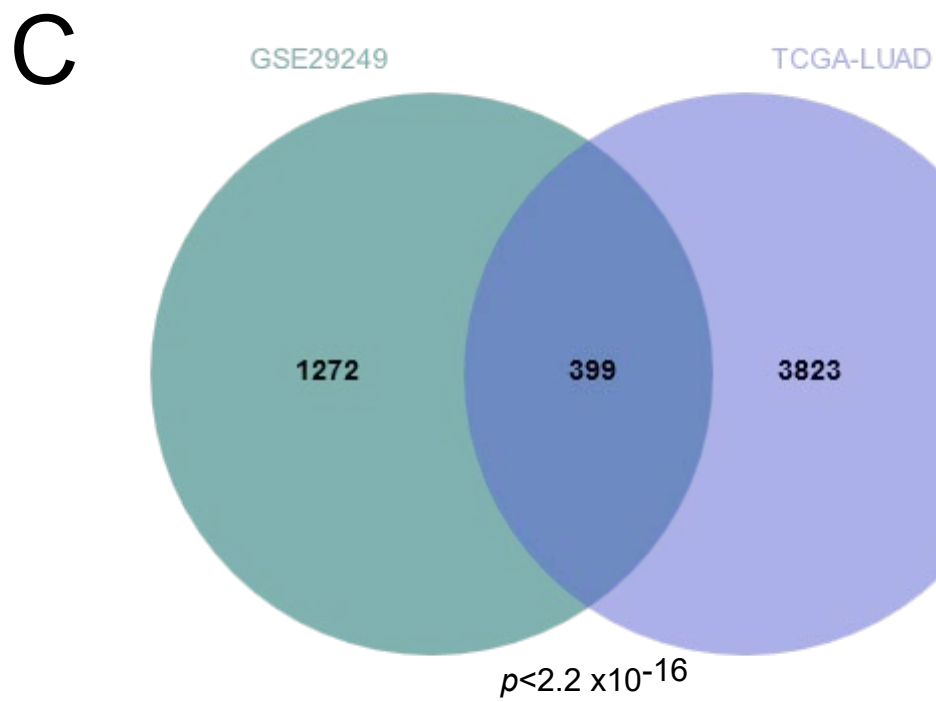

Supplement: Supplementary file 10 — Additional file 10. [file 12885_2021_8462_MOESM10_ESM.pdf]

A

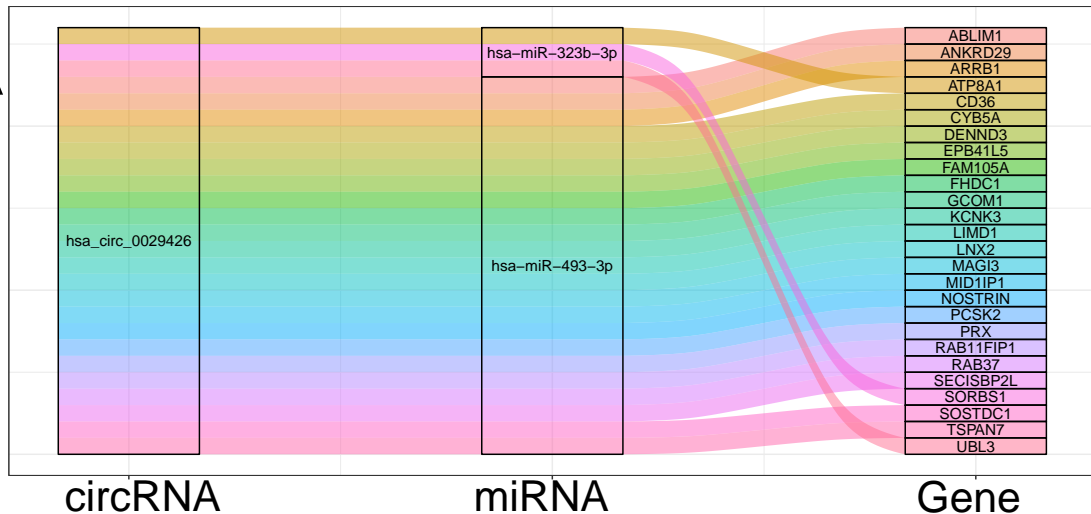

B

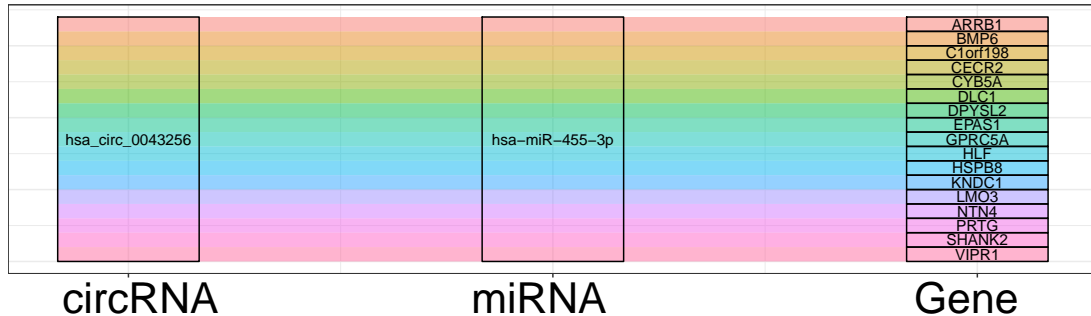

Supplement: Supplementary file 11 — Additional file 11. [file 12885_2021_8462_MOESM11_ESM.pdf]

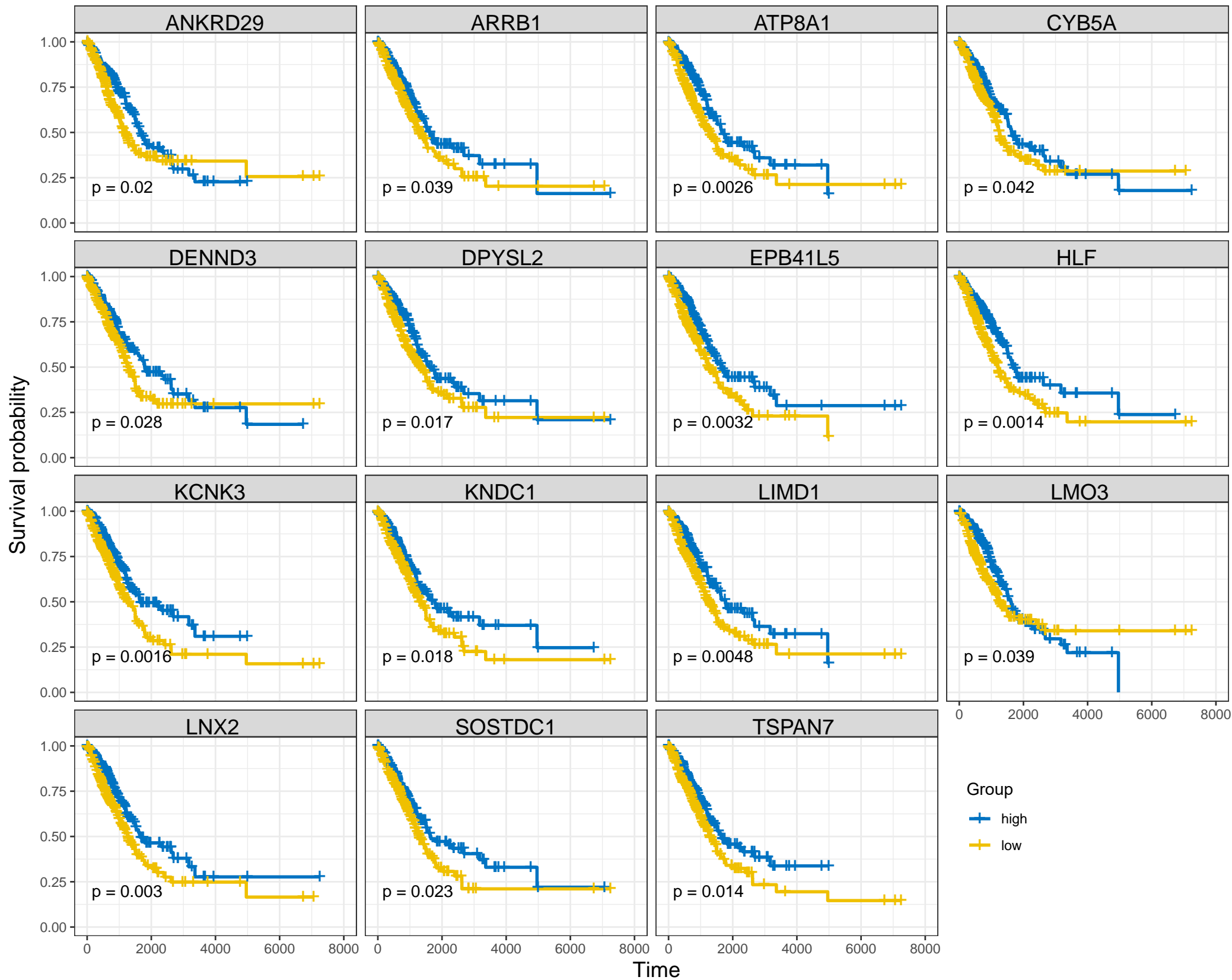

Supplement: Supplementary file 12 — Additional file 12. [file 12885_2021_8462_MOESM12_ESM.pdf]

STAT3 expression level

PDGFB

$R = 0.13, p = 0.0023$

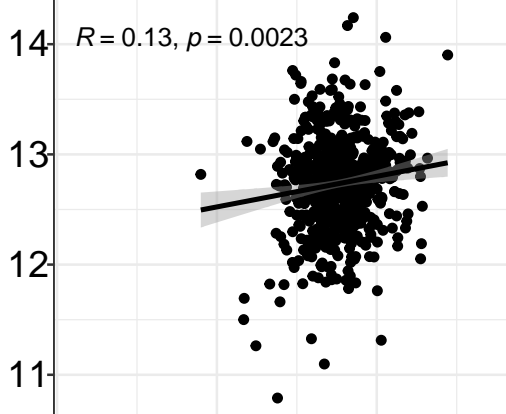

CCND2

$R = 0.23, p = 2.6e-08$

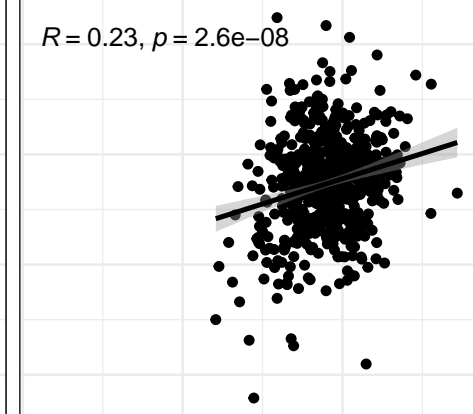

CTF1

$R = 0.17, p = 6.8e-05$

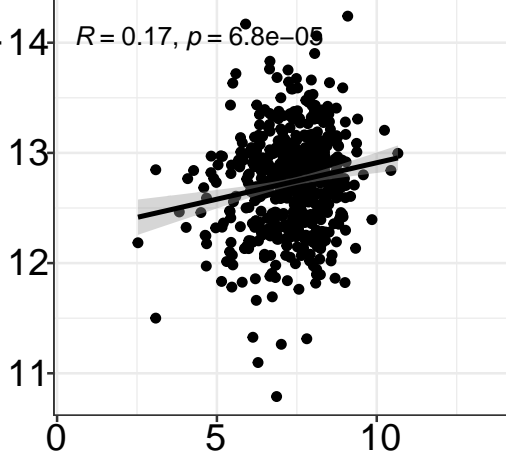

IL7R

$R = 0.25, p = 1e-09$

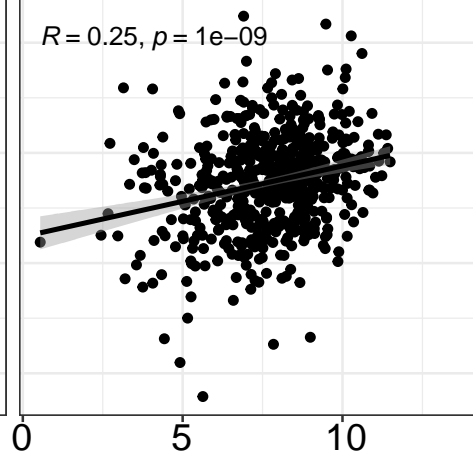

Supplement: Supplementary file 13 — Additional file 13. [file 12885_2021_8462_MOESM13_ESM.pdf]
